# Impact of a referral management “gateway” on the quality of referral letters; a retrospective time series cross sectional review

**DOI:** 10.1186/1472-6963-13-310

**Published:** 2013-08-14

**Authors:** Ally Xiang, Helen Smith, Paul Hine, Katy Mason, Stefania Lanza, Anna Cave, Jonathan Sergeant, Zoe Nicholson, Peter Devlin

**Affiliations:** 1 Division of Primary Care and Public Health, Brighton and Sussex Medical School, Falmer, Brighton BN1 9PH, UK; 2 Brighton and Hove Integrated Care Service Limited, Fourth Floor, 177 Preston Road, Brighton BN1 6AG, UK

**Keywords:** General practice, Referral letters, Quality improvement, Peer review, Referral management

## Abstract

**Background:**

Referral management centres (RMC) for elective referrals are designed to facilitate the primary to secondary care referral path, by improving quality of referrals and easing pressures on finite secondary care services, without inadvertently compromising patient care.

This study aimed to evaluate whether the introduction of a RMC which includes triage and feedback improved the quality of elective outpatient referral letters.

**Methods:**

Retrospective, time-series, cross-sectional review involving 47 general practices in one primary care trust (PCT) in South-East England. Comparison of a random sample of referral letters at baseline (n = 301) and after seven months of referral management (n = 280). Letters were assessed for inclusion of four core pieces of information which are used locally to monitor referral quality (blood pressure, body mass index, past medical history, medication history) and against research-based quality criteria for referral letters (provision of clinical information and clarity of reason for referral).

**Results:**

Following introduction of the RMC, the proportion of letters containing each of the core items increased compared to baseline. Statistically significant increases in the recording of *‘past medical history’* (from 71% to 84%, p < 0.001) and *‘medication history’* (78% to 87%, p = 0.006) were observed. Forty four percent of letters met the research-based quality criteria at baseline but there was no significant change in quality of referral letters judged on these criteria across the two time periods.

**Conclusion:**

Introduction of RMC has improved the inclusion of past medical history and medication history in referral letters, but not other measures of quality. In approximately half of letters there remains room for further improvement.

## Background

Referral letters are a key means of communication between general practitioners (GPs) and hospital specialists. GPs write more than 10 million elective referral letters each year for routine management of conditions [1]. Accumulated evidence suggests that GP referral letters often lack essential clinical information, for example, 38% of specialists in outpatients reported that referral letters contain inadequate information fairly often or very often [2,3]. Letters may fail to explain clearly why the patient is being referred or what is being asked of the specialist team (diagnosis, investigation, treatment or reassurance) [4]. Consequently, GPs, consultants and patients may each have a very different understanding of the purpose of referral. Specialists have said that, in order to better address the patient’s current problem, they would like GPs to provide details that the patient is unable or unlikely to provide themselves, such as detailed past medical history, medications, examination findings, investigation results and details of treatments tried already, as well as more information about the patient’s presenting complaint, the problem to be addressed and clinical questions to be answered [5].

Various methods have been tried to improve letter writing and to produce more informative letters, for example guidelines or standardised referral templates which highlight information preferred by the specialist [5,6]. Used passively and in isolation, guidelines and proformas have not always succeeded in improving the content of referral letters [7,8]. However, multi-faceted interventions such as peer or specialist feedback together with guidelines, proformas or risk factor check-lists have been shown to improve the referral process, including improved letter content [6,9-17]. A Cochrane review of educational, organisational and financial interventions to improve out-patient referral rates and appropriateness has noted that simple interventions and passive dissemination are ineffective in changing clinicians’ behaviour [18].

Referral management schemes (RMS) are recent developments. There is a hierarchy of RMS, from low level interventions, which encourage compliance with guidelines such as the Map of Medicine [19], through to Referral Management Centres (RMCs), where all GP referrals undergo clinical triage. The primary aim of these schemes is to control demand and reduce unnecessary and inappropriate referral of patients to specialist services. However, they also have the potential to influence other aspects of the referral decision and referral process, including the quality of the referral letter.

A 2010 King’s Fund report highlighted a lack of research on the impact of referral management centres, particularly those with centralised models that cover referrals to all specialities. It noted that existing research has not explored the impact of RMC on referral quality [20].

The concerns around poor referral letter quality nationally, and the lack of research on the impact of RMCs prompted this current study. Our aim was to retrospectively assess the introduction of a RMC on the quality of referral letters.

## Methods

**Figure 1:**
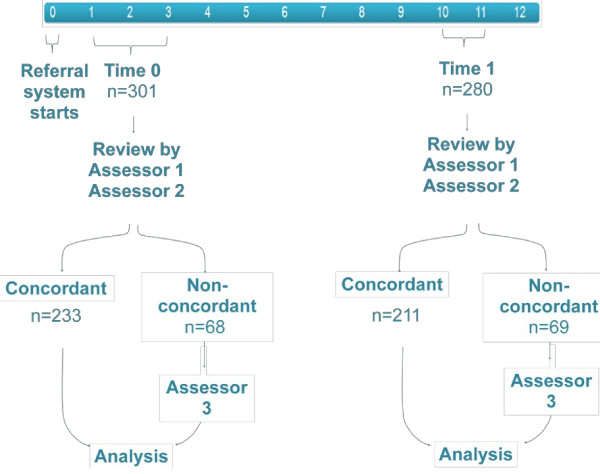
**Sampling and review of referral letters from two time periods.** The non-concordance relates to both objective (explicit) and subjective (implicit) judgements made by assessors 1 and 2 with respect to both sets of quality criteria used.

We studied a RMC established in 2008 for 47 practices (134 whole time equivalent GPs) within one PCT. This centre triages all elective GP referrals (with the exception of maternity, mental health and paediatrics) and directs them to appropriate care providers based on the patient’s perceived needs. If the triaging GP finds the referral letter contains insufficient information or does not adhere to the local management pathways, this is fed back to the referring GP. The feedback is peer to peer, by telephone within three to five days of receipt of the referral. Using a random number generator we identified a samples of 300 referral letters for two time periods: time 0 (a 60 day period one month after the referral management system became fully operational, October-November 2008) and time 1 (a 30 day period seven months later, July 2009) (Figure 1). Letters were reviewed retrospectively and the reviewers were blinded to the date of the letter and the time period from which the letter originated.

The data extracted included the destination speciality of each referral and the letter’s information format (letter only, letter plus selected download of data from electronic patient record, and letter plus unedited additional data).

Two researchers (PH, KM) assessed the content of the referral letters for “quality” using two methods:

•Assessing the presence or absence of four items of information that had been agreed and implemented by the local Practice Based Commissioning board to monitor referral letter quality prior to the introduction of the RMC. These four items considered ‘core’ were blood pressure, body mass index, past medical history and medication history. All GPs had been briefed about the necessity for these items to be included in out-patient referral letters when first introduced as proxy indicators of quality and the triaging GPS were also aware of these indicators.

•Application of the two quality criteria for referral letters developed by Grol and colleagues from national guidelines, international literature and surveys [21]. The referral letter is judged to meet the first criterion (provision of clinical information) when four or more of the following clinical items of information are present: patient symptoms; previous examination findings; whether or not investigations were performed; whether or not treatment had been given; current medication. The second criterion assesses clarity of the reason for referral. To meet this, the letter must contain at least one specific request for the specialist (request for diagnosis, treatment, and/or a management plan). Neither the referring GPs nor the triaging GPs were aware that these criteria would be used to assess referral quality when they wrote the letters.

Letters were reviewed independently and then scores were compared. Where there was non-concordance in the letter quality assessment, a third “expert” assessor (HS) reviewed the letter and the majority decision (two out of three) was used in analysis (Figure 1).

The assessment process and proforma were piloted (n = 20) before commencing the audit and the Kappa values achieved ( >0.7) represented substantial agreement. In this preliminary review, it was recognised that the utility of the data being collected could be improved by capturing some additional variables. Firstly, inclusion of all four items of information (blood pressure, body mass index, past medical history and medication history) was found not to be essential for every referral reviewed. For example, a referral to dermatology about a fast-growing keratising skin lesion that does not include body mass index or blood pressure in the letter may still have sufficient essential information. Therefore the option to comment on relevance of missing data was introduced and reviewers were able to record “not relevant” if data was missing but deemed non-essential to that particular case, or “relevant” if an item was missing but needed.

When piloting the Grol criteria we found several examples where the reason for referral was implied but not stated specifically. In order to explore this poor articulation of the reason for referral further, we categorised each request for the specialist as either ‘explicit’ or ‘implicit’. If a request was judged implicit, the exact phrasing used was noted. As above, we also enabled assessors to make a subjective override of the quality decision based on the assessment criteria. Where there was a majority decision to override the objective decision, these cases were identified in subsequent analyses.

We performed statistical analysis using PASW Statistics 18 (SPSS Inc). Data were nominal and independent, so non-parametric statistics were used. Chi-square tests were used to compare the quality of referral letters between time 0 and 1. Because of the exploratory nature of this study, all analyses were two-tailed, with *p ≤* 0.05 considered significant. As this review was a before and after service evaluation using routinely collected data it did not require ethics committee approval.

## Results

A total of 581 referral letters were reviewed: 301 letters from time 0 (5.1% of the 5902 referrals received) and 280 from time 1 (7.2% of the 3889 referrals received). We were unable to locate 20 of the randomly sampled letters from time 1. The specialities most frequently referred to were orthopaedics (n = 88), gynaecology (n = 83), gastroenterology (n = 74), neurology (n = 72) and dermatology (n = 65). The majority of referral letters (time 0: 196 (65.1%) and time 1: 190 (67.9%)) were in the format of a letter plus selected data downloaded from electronic patient records (Table 1). Seven per cent of referrals were judged by the RMC reviewer to need peer to peer feedback. Feedback was generally verbal, but for those practices using unedited downloads of the electronic patient records to accompany their referral letters, ‘hands on’ administrative support was offered to customise their computer system appropriately.

**Table 1:** Characteristics of referral letters (target specialty, format of correspondence) at time 0 (n = 301) and at time 1 (n = 280)

**Specialty referred to**	**Time 0**	**Time 1**
	**Number (%)**	**Number (%)**
Medical		
Cardiology	11 (3.7)	18 (6.4)
Dermatology	44 (14.6)	21 (7.5)
Endocrinology	7 (2.3)	8 (2.9)
Gastroenterology	37 (12.3)	37 (13.2)
Neurology	43 (14.3)	29 (10.4)
Pain Team	4 (1.3)	11 (3.9)
Respiratory	7 (2.3)	5 (1.8)
Rheumatology	9 (3.0)	12 (4.3)
Other	7 (2.3)	7 (2.5)
Surgical		
Breast	6 (2.0)	7 (2.5)
Ear, nose and throat	0 (0.0)	24 (8.6)
General surgery	7 (2.3)	15 (5.4)
Gynaecology	53 (17.6)	30 (10.7)
Orthopaedics	49 (16.3)	39 (13.9)
Vascular	5 (1.7)	7 (2.5)
Other	12 (4.0)	8 (2.9)
Not stated	0 (0.0)	2 (0.7)
**Format of referral**	**Time 0**	**Time 1**
	**Number (%)**	**Number (%**)
Letter only	82 (27.2)	46 (16.4)
Letter plus selected download of data from electronic patient record	196 (65.1)	190 (67.9)
Letter plus unedited additional data	10 (3.3)	4 (1.4)
Other: examples included referral letters with forwarded radiology result, care plan, copies of previous correspondence, another health professional’s letter, other department investigation results and private clinic letter	13 (4.3)	42 (15.0)

### Inclusion of core information

At both times *‘past medical history’* and *‘medication history’* were recorded in over 70% of letters. *‘Body mass index’* was the least frequently included indicator, in 40.5% letters at time 0 and 43.6% at time 1 (Table 2). 26.9% (81 out of 301) of letters contained all four criteria at time 0, increasing to 38.9% (109/280) at time 1.

**Table 2:** Percentage of referral letters including core information

**Core information**	**Time 0**	**Time 1**	***p***
Blood pressure	48.2%	52.1%	0.38
Body mass index	40.5%	43.6%	0.51
Past medical history	71.2%	84.3%	<0.001
Medications history	77.7%	86.8%	0.006

For all core information items, the proportion of letters containing them increased at time 1 compared to baseline. Increased frequencies in the recording of *‘past medical history’* and *‘medication history’* were statistically significant, but the improvements in inclusion *‘blood pressure’* and *‘body mass index’* did not achieve significance (Table 2).

Of the letters missing one or more items of “core information”, the missing item(s) were judged not essential in 45.1% (152/337) of referrals.

### Grol quality criteria

43.5% referral letters met the Grol quality criteria at time 0 and 39.6% at time 1 (Table 3). The application of the subjective override increased the proportion of letters meeting the criteria to 54.2% and 46.8% respectively, but there was no statistically significant change in the overall quality of referral letters as judged by the Grol quality criteria between the two time periods (Table 3).

**Table 3:** Percentage of referral letters meeting the Grol quality criteria

	**% Meeting criteria**		
	**Time 0**	**Time 1**	**p**
Criterion 1	44.9	41.1	0.40
Criterion 2	94.4	95.0	0.87
Both criteria	43.5	39.6	0.39
After subjective over-ride	54.2	46.8	0.091

Footnote: According to the Grol criteria [20] the referral letter is judged to meet the first criterion (provision of clinical information) when four or more of the following clinical items of information are present: patient symptoms, findings of previous examinations, whether or not investigations were performed, whether or not treatment had been given, and current medication. The second criterion addresses clarity of the reason for referral. To meet this, the letter must contain at least one specific request for the specialist (request for diagnosis, treatment, and/or a management plan).

Examining individual sections within each criterion, *‘patient symptoms’* were the most frequently recorded detail (90.2% over both periods) and *‘whether or not investigations were performed’* least frequent (42.2% overall) (Table 4). Between time 0 and time 1 there were no significant improvements in recording of *‘patient symptoms’*, *‘findings of previous examinations’* or *‘whether or not investigations were performed’*. There was significant improvement in the recording of *‘current medication’* between the two time periods and a significant decline in the recording of *‘whether or not treatment had been given’* between the two periods (Table 4).

**Table 4:** **Number and percentage of referral letters meeting individual sections of the first Grol**[20]**quality criterion**

	**Time 0 number (%)**	**Time 1 number (%)**	***p***
**Patient symptoms**	278 (92.4)	246 (87.9)	0.092
**Findings of previous examinations**	187 (62.3)	156 (55.7)	0.14
**Investigations were performed**	122 (40.5)	123 (43.9)	0.46
**Treatment had been given**	159 (52.8)	111 (39.6)	0.002
**Current medication**	235 (78.1)	244 (87.1)	0.006

The most common request for the specialist within the letter was ‘*request for diagnosis*’, followed by *‘request for management’* and then *‘request for treatment’* (Table 5). In only 39.6% of letters at time 0 and 30.4% at time 1 were these requests explicit (for example: ‘Discuss potential diagnosis of Marfan’, ‘Review and advice regarding further intervention’). In the remaining majority (60.4% at time 0 and 69.6% at time 1) the phraseology used was not explicit. Where the reason for referral was not specific, the reviewers considered whether it could be implied from what had been written, for example ‘assessment of his problem’ or ‘make sure there is nothing serious’ was interpreted as an implicit *‘request for diagnosis’*; ‘deal with this’ and ‘any help’ were considered implicit requests regarding *‘treatment’*; and ‘please advise’ as an implicit request for *‘management advice’*. Using this revision of the criteria over 98% letters could be categorised by type of help being requested of the specialist.

**Table 5:** **Percentage and number of different types of requests contained in referral letters categorised by the second Grol **[20]** quality criterion**

		**Time 0 (n = 301)**			**Time 1 (n = 280)**		
	**Explicit**	**Implicit**	**Total**	**Explicit**	**Implicit**	**Total**	***p***
	**%**	**%**	**% (no.)**	**(%)**	**(%)**	**% (no.)**	
All requests	39.6	58.5	98.1 (295)	30.4	68.3	98.7 (276)	0.785
Specific requests regarding							
-diagnosis	7.3	33.6	40.9 (123)	6.1	40.4	46.5 (130)	0.229
-treatment	17.3	6.3	23.6 (71)	13.9	9.3	23.2 (65)	0.260
-management plan	15.0	18.6	33.6 (101)	10.4	18.6	29.0 (81)	0.240

## Discussion

### Summary of main findings

Our study has demonstrated that since the introduction of a RMC, there has been a significant improvement in some aspects of the quality of the referral letters written by GPs to their specialist colleagues (the inclusion of past medical history and medication history). No improvements were observed using the more demanding Grol criteria which assess the inclusion of more contextual clinical information (whether or not investigations were performed, and treatments had been given) together with formulation of a specific request to the specialist (the provision of a diagnosis and/or treatment and/or management plan).

The limited improvement in referral letter quality found in this study is disappointing. A total of 7% referral letters received peer to peer feedback. Unfortunately the way data were routinely captured did not allow identification of the proportion of feedback that related to the quality of the referral letters. (Feedback could be provided for several other issues, including the appropriateness of referral or target specialty selected). Knowing that at least 25% letters were missing core information relevant to the referral request, higher rates of feedback should have been expected and might have improved referral letter quality over the observation period. Anecdotally, triage staff were reluctant to feedback when they recognised a letter had been written by a locum, as they knew there was little chance of being able to contact that doctor. Better processes are needed to ensure all referrers, irrespective of their employment status, can benefit from peer to peer feedback.

Traditionally an outpatient referral has been considered to represent the transfer of responsibility for some aspect of the patient’s care from primary to secondary care [22]. In referral management a third party is introduced into this referral process. This third party is employed to assess risk, to triage, and to select the most appropriate destination for each patient based on the GP referral letter. Without knowing the patient or having access to their full primary care record, the triaging GP has to depend solely on the contents of the referral letter to inform their decision making. In this situation the quality of the referral letter acquires even greater importance as the absence of key information may lead to erroneous diversion of a referral which would have been judged appropriate if all the relevant information had been available. In future audits, consideration should be given to possible harm to patients that may result from the delay when letters are intercepted and re-routed. We have no evidence to suggest that any patients suffered harm, but our study was not designed to detect this.

### Strengths and limitations of the study

This study tackled an appropriate and important question, addressing the paucity of research identified by the King’s Fund. Furthermore the study has good internal validity, provided by a large, randomly selected sample of letters which are representative of the local referral patterns. The tools used to judge letter quality were piloted before the study to ensure consistency and had acceptable inter-rater reliability (Kappa values >0.7 representing substantial agreement). The risk of bias was further reduced by the use of a third person who independently reviewed any letters where there was a lack of concordance between the two researchers’ assessment. The study has good external validity, as the setting has socio-economically and ethnically diverse population, a variety of practice sizes and a wide range of secondary care services; study findings are thus generalisable to areas within the UK where RMCs may be introduced.

This study has some limitations. It was retrospective, but the risk of this influencing results was reduced by blinding the reviewers to the period from which each letter had been sampled. The credibility of the quality criteria used differed, whilst the Grol quality criteria had been formally developed the four ‘core’ items of information did not have a theoretical base and were adopted as proxy measures of quality. The utility of the four items of information as an outcome measure may be further threatened by GPs’ awareness of these ‘proxy’ quality criteria prior to the introduction of the RMC. The study also lacked any assessment by the receiving specialists of the adequacy of the referral letters or whether they felt the RMC process resulted in any improvements. A further limitation is the relatively short time period between the two assessment periods (seven months) which may not have been long enough for the feedback process to change the practice of individual GPs. That said, there is no research precedent for how long changes based on triage feedback may take and seven months seems a reasonable period for individual GPs to make changes to their practice.

### Comparison with existing literature

We cannot conclude causality, but the introduction of triage and referral management of the referral letters was associated with moderate improvements in some aspects of referral letter quality. Greater shifts in quality have been achieved with more active and multifaceted interventions, for example weekly practice-level referral review meetings and six-weekly cluster meetings including consultant feedback. After one year, that study achieved improved awareness and use of referral guidelines, referral letter content and high acceptability amongst GPs [23].

Nevertheless, it still remains difficult to change the behaviour of an established GP. For example, a qualitative study of GP referrals to psychiatric services concluded that GPs did not use a technical framework to describe patient needs. Instead they described patients’ needs in holistic and social terms, following “reasoned recipes of action indicating how to bring forth typical results in typical situations by typical means”. The letters reflected the strength of practitioner’s sense of acting as a gateway to secondary services, the degree of suffering, disruption or perceived urgency. Interestingly, GPs expressed unwillingness to make clear, explicit requests for a particular form of support or treatment, which was attributed to a traditional deference to “expert help” rather than true deference [7]. These observations may help explain our observation that GPs frequently failed to make explicit requests regarding diagnosis, treatment or management plans.

### Implications for future research or clinical practice

The poor quality of out-patient referral letters is a persistent problem and whilst this study suggests a potential role for a referral management gateway to contribute to improvement there remains a need for further research. Evaluation over a longer period is necessary to assess its full potential and whether impact can be sustained. This preliminary evaluation has highlighted the importance of refining the intervention. For example, the future formal training of triaging GPs in peer to peer feedback may enhance their impact. The documentation on feedback could provide both qualitative data to inform training needs but also the design of more inclusive feedback processes that enable all referrers, irrespective of their employment status, to benefit.

Previous studies have observed an unwillingness amongst GPs to make a clear explicit statement about the reason for referral, this evaluation also confirms this reluctance. We observed an unfortunate trend with the introduction of referral management towards fewer letters including an explicit statement about the reason for referral. This greater vagueness about reason for referral may be an unintended consequence of referral management, arising as referrers learn to ‘hedge their bets’ to minimise the risk of a referral being deflected or refused by the RMC. To overcome this behaviour change, a within-practice intervention may be helpful, such as internal peer review prior to sending the letter, which uses a checklist based on the Grol quality criteria for explicit requests.

GP training, continuous professional training, and revalidation should all include development of letter writing skills. For example, paediatric specialty training in the UK utilises the Sheffield Assessment Instrument for Letters (SAIL) [24]. This is an 18-point check-list covering areas such as problem list, history, examination, overall assessment, management, follow-up and clarity, and a 10-point scale to record a global rating of the letter against gold standard, which was defined as ‘This letter clearly conveys the information I would like to have about the patient if I were the next doctor to see him/her’. Individualised feedback is then relayed to the writer to enable reflection and self improvement. SAIL has been shown to be effective in improving the quality of outpatient letters to GPs [25].

## Conclusions

This study begins to address the evidence gap highlighted by the 2010 King’s Fund report. With the increasing importance of RMCs, more research is needed to address their efficacy and usefulness. By improving the quality of referral letters, decisions on the appropriateness of referrals are likely to become more accurate and specialists will be provided with the information they need to complement the patient’s own history and reach a better management plan, making their part of the process more efficient and effective.

## Abbreviations

GP: General practitioner; PASW: Predictive analytics SoftWare; PCT: Primary care trust; RMC: Referral management centre; RMS: Referral management scheme; SAIL: Sheffield assessment instrument for letters; SPSS: Statistical package for the social sciences.

## Competing interests

Prof Helen Smith is a shareholder of Brighton & Hove Integrated Care Services (BICS) Limited. Dr Jonathan Serjeant, Zoe Nicholson and Dr Peter Devlin are Executive Directors of BICS Limited.

## Authors’ contributions

HS & SL devised the project with JS, ZN and PD. The extraction and anonymisation of referral letters was co-ordinated by SL. AX, PH, KM, AC and HS reviewed the referral letters, and entered data. AX, KM and PH analysed the data and drafted the first manuscript under HS’s supervision. All authors contributed to the interpretation of data and refinement of the manuscript. All authors read and approved the final manuscript.

## Pre-publication history

The pre-publication history for this paper can be accessed here:

http://www.biomedcentral.com/1472-6963/13/310/prepub
